# The usefulness of narrow band imaging in the assessment of laryngeal papillomatosis

**DOI:** 10.1371/journal.pone.0205554

**Published:** 2018-10-09

**Authors:** J. Jackowska, H. Klimza, P. Winiarski, K. Piersiala, M. Wierzbicka

**Affiliations:** 1 Department of Otolaryngology, Head and Neck Surgery, Poznań University of Medical Sciences, Poznań, Poland; 2 Department of Otolaryngology, Otolaryngological Oncology and Maxillofacial Surgery, Uniwersytet Mikolaja Kopernika Collegium Medicum, Bydgoszcz, Poland; 3 Student Research Group at the Department of Otolaryngology, Head and Neck Surgery, Poznań University of Medical Sciences, Poznań, Poland; University of Bern, University Hospital Bern, SWITZERLAND

## Abstract

**Objectives/Hypothesis:**

Recurrent respiratory papillomatosis (RRP) is a benign disease caused by human papillomavirus 6 and 11. The characteristic feature of this disease are wart-like lesions covering the respiratory epithelium with a predilection for the larynx. There is no curative treatment for the disease. The goal of the treatment is a total surgical removal of the papillomatous lesions in order to reduce the number of relapses. Therefore, a good visualization method of papillomas is crucial during surgery. The aim of the study was to compare the accuracy of narrow band imaging (NBI) to the use of white light alone in detecting RRP.

**Methods:**

The study was carried out between April 2013 and November 2015 at Poznan University of Medical Sciences, Department of Otolaryngology, Poland. Rigid endoscopy with conventional white light (WL) and NBI (CV-260SL processor and CLV- 260SL light source, Olympus Optical Co. Ltd, Tokyo, Japan) was performed in all patients during direct laryngoscopy. All anatomical sites of the larynx and trachea were assessed using the Dikkers scale and Derkay total site scoring system with WL and NBI. The consensus was reached as to the number of lesions seen in WL compared to NBI.

**Results:**

During 36 microlaryngoscopies, the number of papillomas detected in the larynx (by Derkay total site score) differed significantly between white light endoscopy and NBI (Wilcoxon test p = 0.000655). In endoscopy with NBI, a mean of 1.3 more papillomas in Derkay total site score was detected in comparison to white light endoscopy NBI showed additional areas of diseased tissue in 15/36 (41.67%) patients.

**Conclusions:**

NBI as an additional tool during microlaryngoscopy can improve the detection of papillomatous lesions.

## Introduction

Recurrent respiratory papillomatosis (RRP) is a rare (with an incidence of 4.3 per 100 000 among children and 1.8 per 100 000 among adults [1]), benign disease caused by human papillomavirus (HPV) 6 and 11[2]. Papillomas may spread along the entire surface of the respiratory tract, demonstrating a significant predilection for the larynx. Surgical removal remains the first-line treatment method. The goal of RRP therapy is a precise surgical removal of every single lesion. However, even despite a positive outcome of the surgery, laryngeal papillomatosis has a high rate of recurrence and currently, there is no curative treatment for the disease. Some patients may require over 100 surgeries for complete removal of papillomata [3]. Frequently recurring lesions may lead to scarring of the vocal folds. As a result, patients may suffer from lower voice quality. According to Loizu et al. 68 to 78% of adult patients suffering from RRP experience voice dysfunction [4].

The current treatment protocols for RRP include surgery, aiming removal of epithelial lesions while maintaining the underlying anatomical structure of the vocal folds [5]. Intraoperatively, it is often challenging to locate all superficial papillomata, especially in the case of a diffuse disease. Based on the experience of our department, these lesions often remain undiagnosed during microlaryngoscopy due to their small size.

Therefore, we examined the usefulness of narrow band imaging (NBI) in the intraoperative assessment of patients suffering from RRP, analyzed whether this method could help to identify additional RRP lesions during surgery and compared the positive predictive value of NBI vs. white light alone in detecting RRP.

Narrow band imaging (NBI) is a novel optical technique that selects the wavelengths of white light with two peaks around 415 and 540 nm. These wavelengths penetrate only into the superficial layer of mucosa and are absorbed by haemoglobin in capillary vessels. The technology allows identification of vascular patterns that are invisible during white light endoscopy and facilitates identification of superficial capillaries and neoangiogenesis in the abnormal mucosa. The most widely used classification to describe pre- and cancerous lesions of mucosa in NBI examination was presented by Ni et al. [6]. NBI may be also useful in patients suffering from RRP, as by enhancing the contrast between mucosal epithelium and submucosal vessels, NBI highlights pathological vessels in precancerous and cancerous lesions, along with benign lesions such as laryngeal papillomatosis [7, 8].

## Material and methods

The prospective study was carried out between April 2013 and November 2015 in a tertiary referral hospital at Poznan University of Medical Sciences, Department of Otolaryngology, Poland. The study included patients with suspected papilloma-like lesions and those who had a laryngeal papillomatosis confirmed in histology before. All patients underwent routine microlaryngoscopy under general anaesthesia. There were no exclusion criteria.

**Fig 1:**
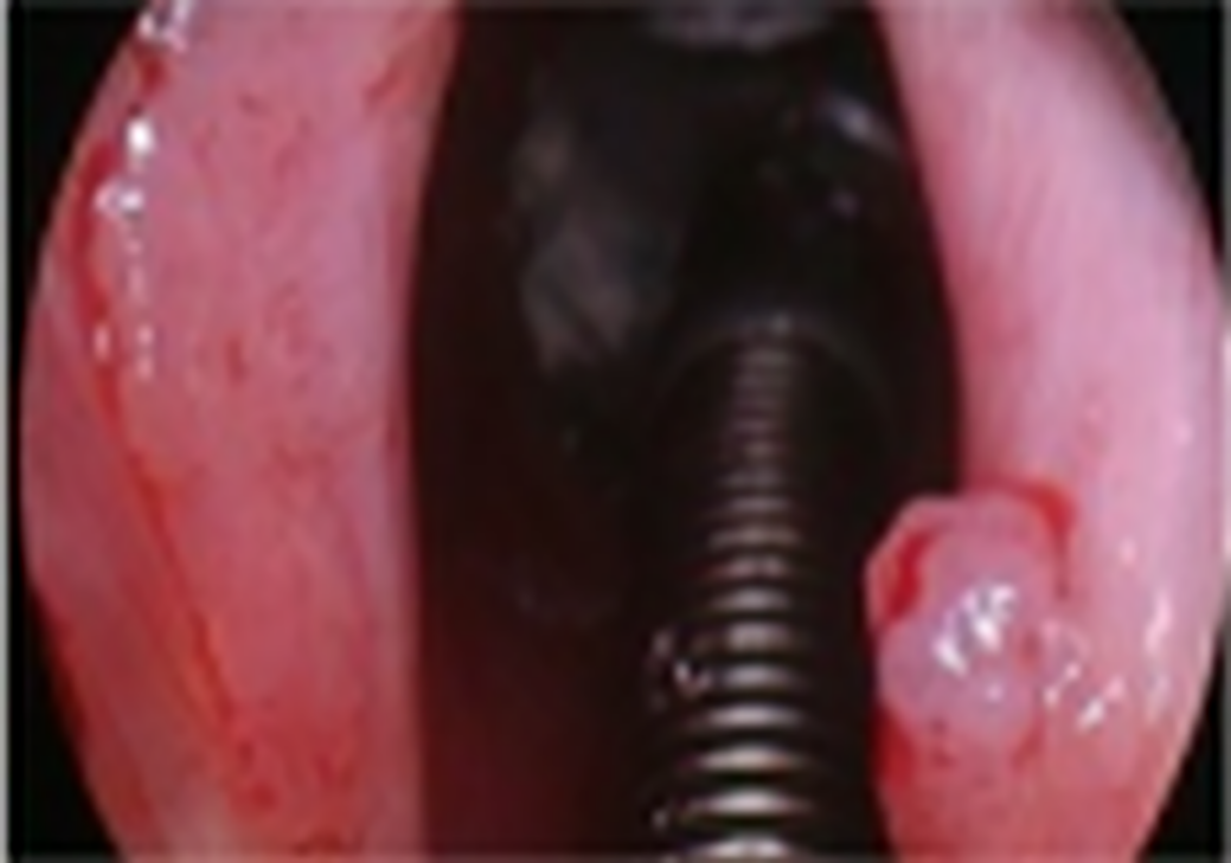
Laryngeal papillomatosis in white light (WL).

**Fig 2:**
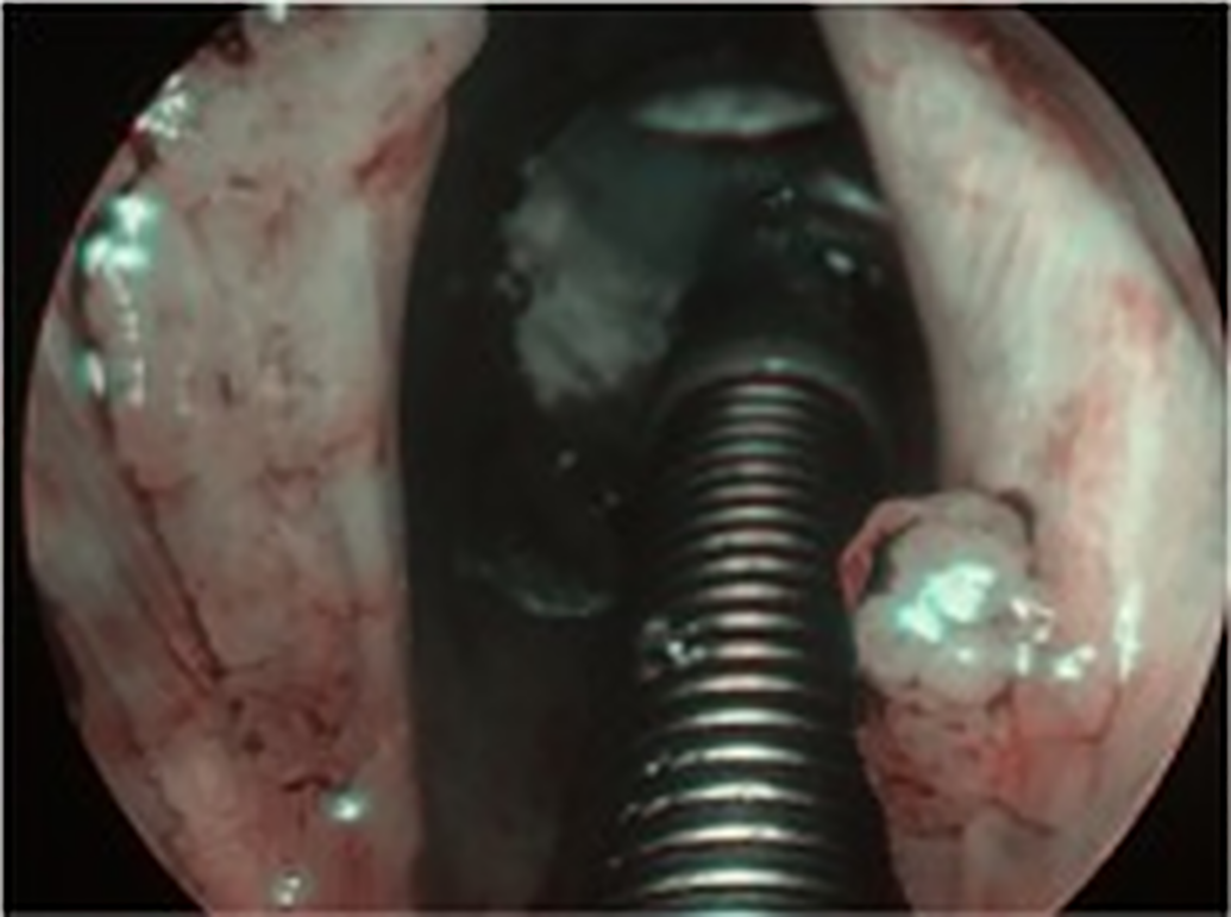
Laryngeal papillomatosis in NBI.

**Fig 3:**
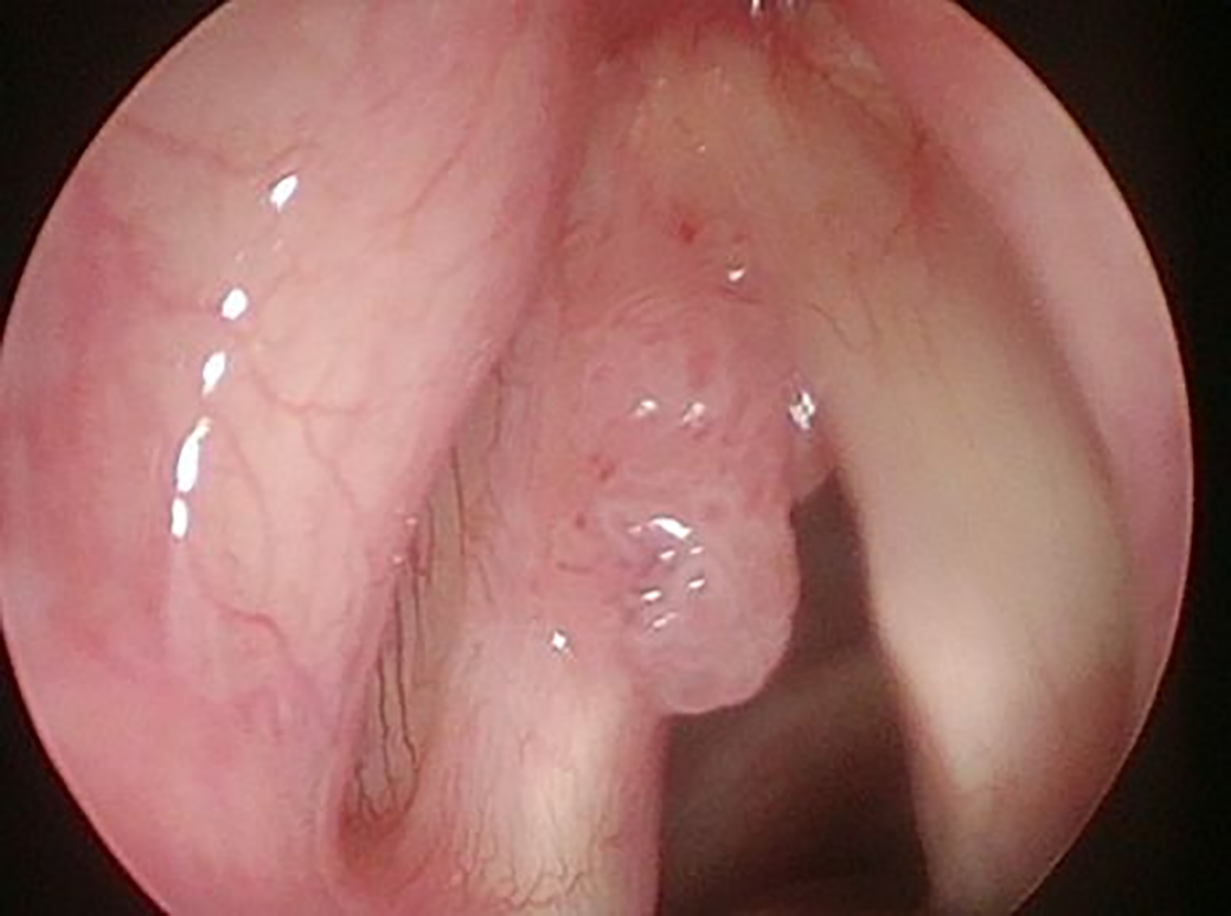
Laryngeal papillomatosis in white light (WL).

**Fig 4:**
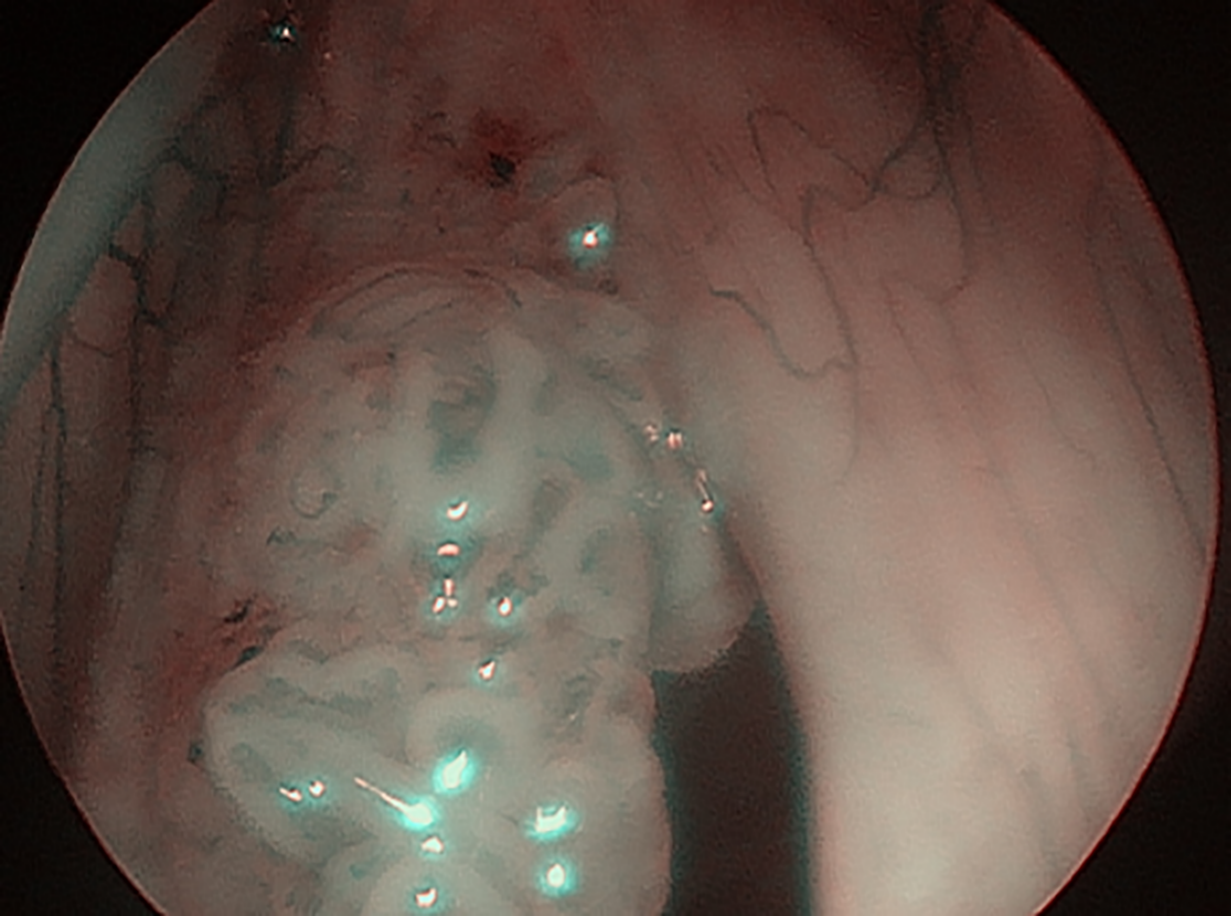
Laryngeal papillomatosis in NBI with vessels along the central axis of each papilla.

Thirty-six (36) consecutive patients, 21 (58.3%) males and 15 (41.67%) females, aged 11–78 years (mean age of 47.31) were enrolled. Two out of 36 patients declared regular tobacco smoking, 8/36 declared being occasional alcohol drinkers. None of the patients had history of malignancies in the past. Laryngeal papillomatosis was histologically confirmed in all lesions. The patients had undergone 0–119 surgical procedures (mean 17.81) before inclusion in the study. Rigid endoscopic examination with white light (WL) and NBI (CV-260SL processor and CLV- 260SL light source, Olympus Optical Co. Ltd, Tokyo, Japan) was performed in all patients during direct laryngoscopy. At first, all anatomical sites of the larynx and trachea were endoscopically visualized with white light and assessed using the Dikkers scale [9]. Afterwards, the anatomic borders of RRP-as were assessed using the modified Derkay staging system and examined with WL and NBI, Figs 1 and 2. In the presented study, only the total site score of Derkay severity scale was used to assess the enrolled patients [10]. Endoscopic evaluation was performed by two experienced head and neck surgeons (JJ, HK). Each part of the larynx (epiglottis, lingual surface, laryngeal surface, aryepiglottic folds, false vocal folds, true vocal folds, arytenoids, anterior commissure, posterior commissure, subglottic) and trachea (upper one-third, middle one-third, lower one-third, bronchi: right, left, tracheotomy) were assessed for the presence of: 1: surface lesion, 2: raised lesion, or 3: bulky lesion. At the end of the endoscopic examination with white light and NBI, accuracy in detecting laryngeal papillomatosis using the modified Derkay scale was compared. According to the current literature, RRP in NBI can be characteristically detected as regular multiple papillae with vessels along the central axis of each papilla [11]. Therefore, during NBI examination, we focused on the central axis of each papilla, Figs 3 and 4. After endoscopic examination with WL and NBI, surgery was performed using a microdebrider and CO2 laser vaporization. Eventually, surgical specimens were sent for routine histopathological examination.

Informed consent: Informed written consent was obtained from all individual participants included in the study.

Ethical approval: All procedures performed in studies involving human participants were in accordance with the ethical standards of the institutional and national research committee and with the 1964 Helsinki declaration and its later amendments or comparable ethical standards. The study protocol was approved by Bioethics Committee of Poznan University of Medical Sciences.

### Statistical analysis

The Wilcoxon test was conducted to compare the results obtained with WL and NBI. The chi-squared test was used to test the difference in number of papillomas according to the Derkey total site scoring system with NBI versus WL. Correlation between age, number of procedures and Derkay total site score in endoscopy with WL and NBI was analyzed using the Kruskal-Wallis test. All tests were conducted at the significance level of 0.05. All of the analyses were performed using Statistica version 12.0 (StatSoft Polska) and Excel 2007 (Microsoft Corp.).

## Results

**Fig 5:**
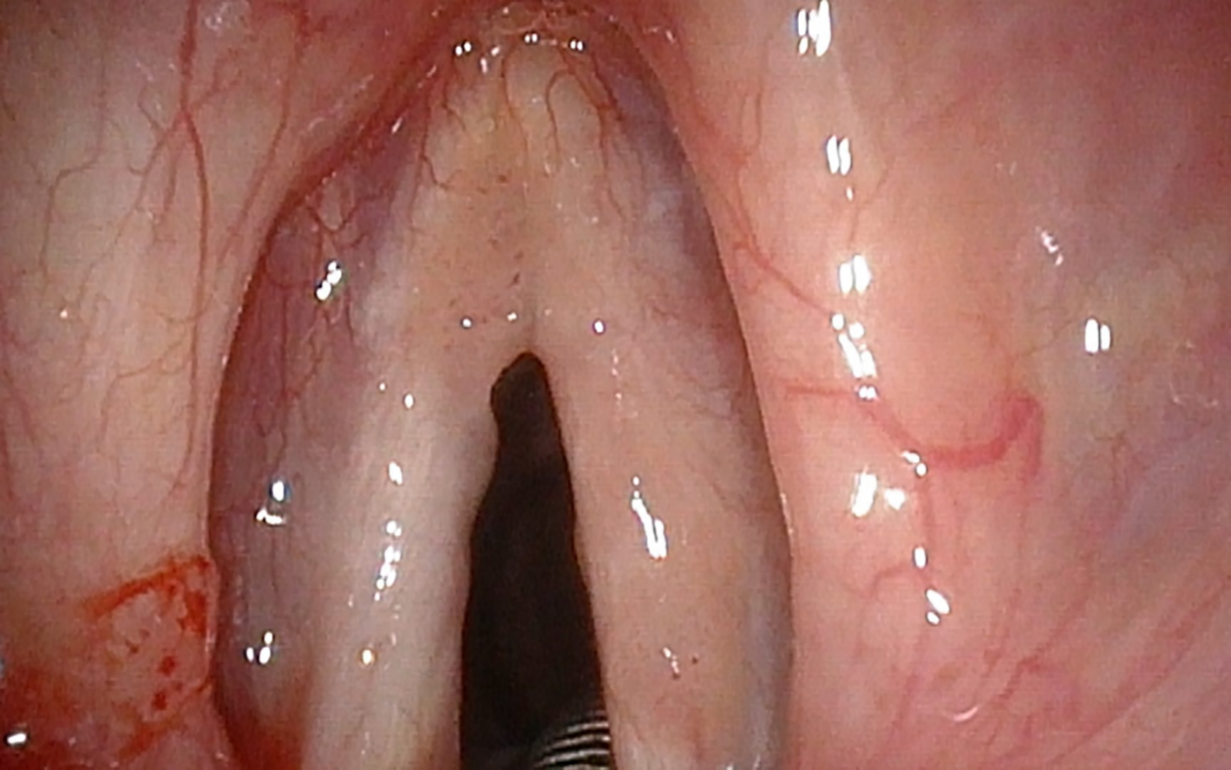
Laryngeal papillomatosis invisible in white light (WL).

**Fig 6:**
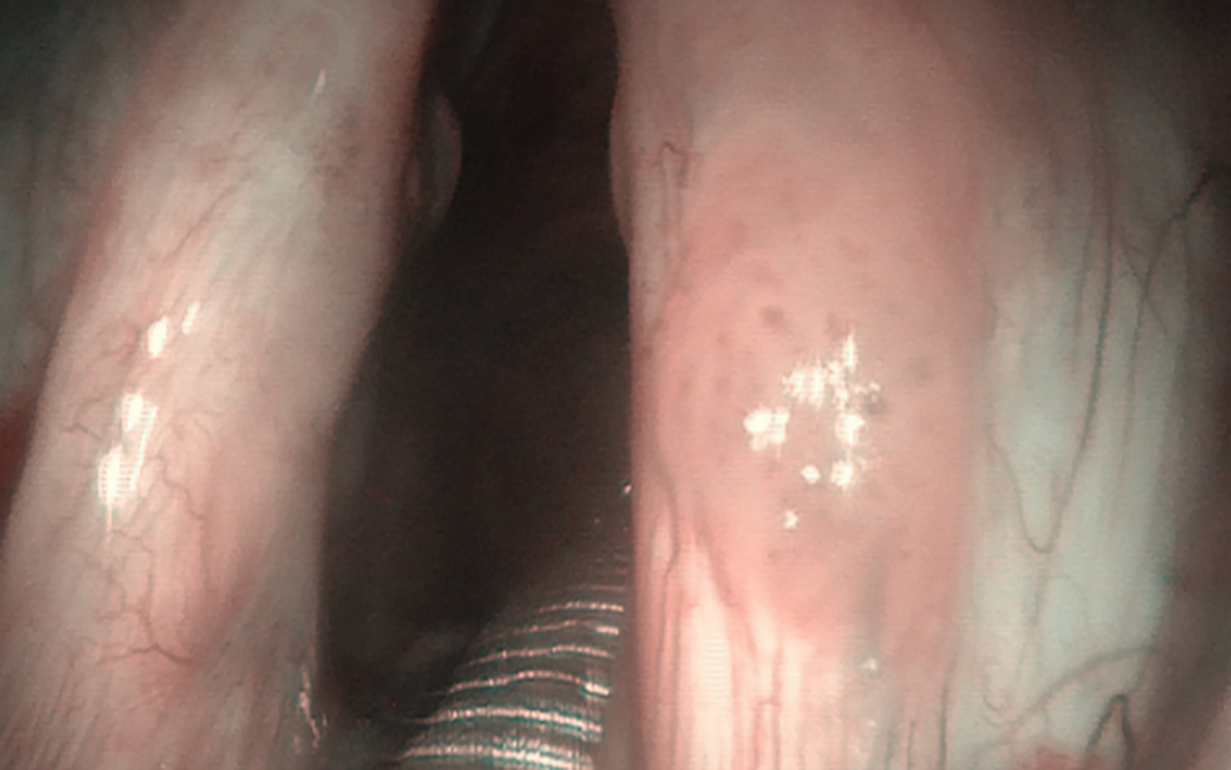
Laryngeal papillomatosis visible in narrow-band imaging (NBI).

The Derkay total site score of papillomas detected in the larynx differed significantly between endoscopy with WL and NBI (Wilcoxon test, p = 0.000655). In endoscopy with NBI, a 1.3 point increase in Derkay total site score was detected compared to WL endoscopy (Table 1), Figs 5 and 6. All lesions were histologically assessed and confirmed by a dedicated pathologist from the Department of Pathology at Poznan University of Medical Sciences. NBI showed additional areas of diseased tissue in 15/36 (41.67%) patients. The number of additional samples in all patients was 26 (laryngeal ventricle: 5/26 (19.24%), false vocal fold: 10/26 (38.46%), true vocal fold: 3/26 (11.54%), arytenoid: 2/26 (7.69%), aryepiglottic: 2/26 (7.69%), subglottic: 1/26 (3.85%), trachea: 1/26 (3.85%), anterior commissure: 1/26 (3.85%), posterior commissure: 1/26 (3.85%).

**Table 1:** Characteristic of patients with confirmed RRP treated by CO2 laser.

patient ID	№ of previous procedures	Dikkers score	WL endoscopy (Derkay total site score)	NBI (Derkay total site score)	Exact location of additional lesions detected by NBI
1.	54	3	10	14	Subglottic + trachea
2.	24	1	2	4	True vocal folds
3.	4	3	4	4	-
4.	1	2	2	2	-
5.	3	3	4	8	False vocal folds
6.	1	1	4	8	Laryngeal ventricle, Anterior commisure
7.	8	1	4	4	-
8.	1	2	2	2	-
9.	15	1	6	10	Laryngeal ventricle
10.	3	3	4	4	-
11.	8	3	2	6	False vocal folds
12.	119	1	6	12	Aryepiglottic, false vocal folds
13.	15	3	10	10	
14.	10	3	4	4	
15.	2	3	4	4	
16.	9	1	4	4	-
17.	4	1	4	6	False vocal fold
18.	5	1	4	8	Aryepiglottic False vocal fold
19.	30	3	6	6	
20.	1	1	4	6	Arytenoid
21.	20	3	4	4	-
22.	2	3	10	10	
23.	3	1	8	10	True vocal fold
24.	7	3	10	14	Arytenoid, false vocal folds
25.	106	3	10	10	-
26.	0	2	2	2	-
27.	1	2	2	2	-
28.	2	2	2	2	-
29.	48	2	2	2	-
30.	40	3	4	4	-
31.	1	2	4	4	-
32.	3	1	2	4	Posterior commisure
33.	1	2	2	2	-
34.	0	1	6	8	Laryngeal ventricle
35.	90	1	4	6	Laryngeal ventricle
36.	1	1	2	2	-
Total site score	-	-	164	212	-

There was a statistically significant correlation between the Dikkers score and additional lesions detected with NBI (Chi2, p = 0.00069). Additional papillomas were visualized in NBI in 11/14 (78.57%) patients with sessile lesions (unifocal or multifocal) scoring 1 on the Dikkers scale. No additional papillomas were detected in NBI among patients with exophytic papillomas scoring 2 on the Dikkers scale, however in 4/14 (28.57%) patients with exophytic papillomas scoring 3 on the Dikkers scale there were additional lesions found in NBI.

There was no significant correlation between age, number of procedures and Derkay total site score in endoscopy with white light and NBI (Kruskal-Wallis test, p >0.05).

There was also no statistically significant correlation between age and assessment of lesions using the Dikkers scale (Kruskal-Wallis test, p = 0.9600).

The analysis confirmed that there is no statistically significant correlation between gender and assessment of lesions using the Dikkers scale (Chi2, p = 0.28680). Recurrence of papillomas was observed in 1/36 patients within 12 months after the operation.

## Discussion

In the presented paper, we focused on the intraoperative use of narrow-band imaging (NBI) in patients suffering from RRP. NBI has been characterized by high sensitivity, specificity, negative predictive value and positive predictive value in the detection of precancerous and cancerous lesions of head and neck region in several studies [12,13,14]. However, there have been no previous reports of the intraoperative use of NBI in a significant group of RRP patients. Therefore, we decided to summarize and systematize the comparison of these two techniques—NBI and white light endoscopy, in the assessment of papilloma lesions.

The first study showing increased sensitivity of NBI in the detection of RRP lesions compared with conventional WL alone was presented by Tjon Pian et al. [15]. Authors demonstrated that the sensitivity and specificity of WL with accompanying NBI were 97% and 28%, respectively. Nearly seventy-seven percent (77%) of lesions suspected of LP and biopsied under NBI were proven to be accurately diagnosed according to pathology results. They confirmed that NBI is a useful diagnostic method of recurrent respiratory papillomatosis [15].

Ochsner and Klein reported the use of NBI in the treatment of laryngeal papillomatosis in awake patients. They concluded that NBI improved the ability to visualize diseased tissue in 90.0% of patients and showed additional areas of diseased tissue in 46.7%. The most interesting observation was the role of NBI in determining the borderline between papillomas and healthy tissue. NBI defined the borders of disease more clearly in as much as 76.7% of all patients [16]. The authors suggest that the use of flexible endoscopy with NBI before surgery may limit the number of surgical procedures. According to their opinion, NBI has a value in treating patients with RRP in the outpatient setting, which may lead to improved outcomes and thus, reduce the economic costs of treatment. Imaizumi et al. proved that NBI's assistance in identifying the lesion borders facilitated a more effective papilloma removal using CO2 laser ablation or microdebrider excision [17].

According to our results, NBI endoscopy was highly useful in visualizing superficial papillomas as well as lesions located in the false vocal folds and laryngeal ventricle. Although we consider NBI to be easy to use (one button to switch between WL and NBI), we have also realized that NBI has some limitations. First of all, the learning curve for recognizing and differentiating lesions is a long-lasting process. The longer the method is used to visualize epithelial vessels, the easier it is to recognize the characteristic pattern of vessels corresponding to papillomas [11, 14]. Secondly, the light wavelength used in NBI is captured by haemoglobin, therefore even minor bleeding makes it impossible to accurately assess the larynx epithelium. In our opinion, a good co-operation with the anesthesiologist, gentle intubation under eye control and a narrow lumen of endotracheal tube are crucial in order to prevent epithelial damage during intubation, which makes it impossible to use NBI. We admit that our study has a number of limitations, including conducting it in one institution as well as having a small sample size and heterogeneity of the presented group (children and adults). Our research reveals that the use of NBI facilitates distinguishing between papillomas and healthy tissue. However, we must remember that there are a few limitations to this method, such as lack of objective evaluation and lack of efficacy in case of bleeding. In our opinion, a long-term study using NBI during the treatment and postoperative follow-up of patients with laryngeal papillomatosis are necessary to prove its true benefit in preventing RRP recurrence. Nevertheless, our study suggests that NBI technology has a significant value in treating patients with RRP.

## Conclusions

In our opinion, the most important aspect of treatment of RRP is visualizing even very small laryngeal papillomas for complete surgical removal. Using NBI as an additional tool during endoscopy improved the detection of these lesions in the current study.

## References

[1] Armstrong LR, Derkay CS, Reeves WC. Initial results from the national registry for juvenile-onset recurrent respiratory papillomatosis. RRP Task Force. Arch Otolaryngol Head Neck Surg. 1999;125: 743–8. 10406310 10.1001/archotol.125.7.743

[2] Hughes OR. Toward prevention or cure for recurrent respiratory papillomatosis. Laryngoscope 2012;122(suppl 4):S63–S64. 23254606 10.1002/lary.23805

[3] Derkay CS, Wiatrak B. Recurrent respiratory papillomatosis: a review. Laryngoscope 2008 7;118(7):1236–47. 10.1097/MLG.0b013e31816a7135 18496162

[4] Loizou C, Laurell G, Lindquist D, Olofsson K. Voice and quality of life in patients with recurrent respiratory papillomatosis in a northern Sweden cohort. Acta Otolaryngol 2014;134:401–06. 10.3109/00016489.2013.867457 24433057

[5] Adachi K, Umezaki T, Kiyohara H, Komune S. New technique for laryngomicrosurgery: narrow band imaging-assisted video-laryngomicrosurgery for laryngeal papillomatosis. J Laryngol Otol. 2015;129: S74–S76. 10.1017/S0022215114002436 25706167

[6] Ni X-G, He S, Xu Z-G, Gao L, Lu N, Yuan Z, et al Endoscopic diagnosis of laryngeal cancer and precancerous lesions by narrow band imaging. J Laryngol Otol. 2011;125: 288–296. 10.1017/S0022215110002033 21054921

[7] Dippold S, Becker C, Nusseck M, Richter B, Echternach M. Narrow band imaging: A tool for endoscopic examination of patients with laryngeal papillomatosis. Ann Otol Rhinol Laryngol 2015 11;124(11):886–92. 10.1177/0003489415590656 Epub 2015 Jun 8. 26056395

[8] Dippold S, Nusseck M, Richter B, Echternach M. The use of narrow band imaging for the detection of benign lesions of the larynx. Eur Arch Otorhinolaryngol 2017 2;274(2):919–23. 10.1007/s00405-016-4300-2 Epub 2016 Sep 8. 27631509

[9] Dikkers FG. Treatment of recurrent respiratory papillomatosis with microsurgery in combination with intralesional cidofovir—a prospective study. Eur Arch Oto-Rhino-Laryngology. 2006;263: 440–443. 10.1007/s00405-005-1013-3 16328406

[10] Derkay CS, Malis DJ, Zalzal G, Wiatrak BJ, Kashima HK, Coltrera MD. A staging system for assessing severity of disease and response to therapy in recurrent respiratory papillomatosis. Laryngoscope. 1998;108: 935–7. 9628513 10.1097/00005537-199806000-00026

[11] Lukes P, Zabrodsky M, Lukesova E, Chovanec M, Astl J, Betka A. J, et al The role of NBI HDTV magnifying endoscopy in the prehistologic diagnosis of laryngeal papillomatosis and spinocellular cancer. BioMed Research International: Volume 2014, Article ID 285486, 7 pages. 10.1155/2014/285486 PMC4083210 25025043

[12] Piazza C, Cocco D, De Benedetto L, Del Bon F, Nicolai P, Peretti G. Narrow band imaging and high definition television in the assessment of laryn- geal cancer: a prospective study on 279 patients. Eur Arch Otorhinolaryngol 2010;267:409–14. 10.1007/s00405-009-1121-6 19826829

[13] Klimza H, Jackowska J, Tokarski M, Piersiala K, Wierzbicka M. Narrow-band imaging (NBI) for improving the assessment of vocal fold leukoplakia and overcoming the umbrella effect Maitland KC, editor. PLoS One. Public Library of Science; 2017;12: e0180590 10.1371/journal.pone.0180590 28662209 PMC5491250

[14] Piazza C, Cocco D, De Benedetto L, Del Bon F, Nicolai P, Peretti G. Narrow band imaging and high definition television in the assessment of laryngeal cancer: a prospective study on 279 patients. Eur Arch Oto-Rhino-Laryngology. 2010;267: 409–414. 10.1007/s00405-009-1121-6 19826829

[15] Tjon Pian Gi RE, Halmos GB, van Hemel BM, van den Heuvel ER, van der Laan BF, Plaat BE, et al Narrow band imaging is a new technique in visualization of recurrent respiratory papillomatosis. Laryngoscope 2012 8;122(8):1826–30. 10.1002/lary.23344 Epub 2012 May 7. 22566012

[16] Ochsner MC, Klein AM. The utility of narrow band imaging in the treatment of laryngeal papillomatosis in awake patients. J Voice 2015 5;29(3):349–51. 10.1016/j.jvoice.2014.08.002 Epub 2015 Feb 11. 25682190

[17] Imaizumi M, Okano W, Tada Y, Omori K. Surgical treatment of laryngeal papillomatosis using narrow band imaging. Otolaryngol Head Neck Surg 2012 9;147(3):522–4. 10.1177/0194599812448162 Epub 2012 May 17. 22597574

